# The alteration of gray matter volume and cognitive control in adolescents with internet gaming disorder

**DOI:** 10.3389/fnbeh.2015.00064

**Published:** 2015-03-20

**Authors:** Hongmei Wang, Chenwang Jin, Kai Yuan, Tahir Mehmood Shakir, Cuiping Mao, Xuan Niu, Chen Niu, Liping Guo, Ming Zhang

**Affiliations:** ^1^Department of Medical Imaging, The First Affiliated Hospital of Medical College, Xi’an Jiaotong UniversityXi’an, China; ^2^School of Life Science and Technology, Xidian UniversityXi’an, China; ^3^Engineering Research Center of Molecular and Neuro Imaging Ministry of EducationXi’an, China

**Keywords:** internet addiction disorder, gray matter, cognitive control, anterior cingulate cortex, color-word stroop

## Abstract

**Objective**: Internet gaming disorder (IGD) has been investigated by many behavioral and neuroimaging studies, for it has became one of the main behavior disorders among adolescents. However, few studies focused on the relationship between alteration of gray matter volume (GMV) and cognitive control feature in IGD adolescents.

**Methods**: Twenty-eight participants with IAD and twenty-eight healthy age and gender matched controls participated in the study. Brain morphology of adolescents with IGD and healthy controls was investigated using an optimized voxel-based morphometry (VBM) technique. Cognitive control performances were measured by Stroop task, and correlation analysis was performed between brain structural change and behavioral performance in IGD group.

**Results**: The results showed that GMV of the bilateral anterior cingulate cortex (ACC), precuneus, supplementary motor area (SMA), superior parietal cortex, left dorsal lateral prefrontal cortex (DLPFC), left insula, and bilateral cerebellum decreased in the IGD participants compared with healthy controls. Moreover, GMV of the ACC was negatively correlated with the incongruent response errors of Stroop task in IGD group.

**Conclusion**: Our results suggest that the alteration of GMV is associated with the performance change of cognitive control in adolescents with IGD, which indicating substantial brain image effects induced by IGD.

Adolescence is a particular developmental period with rapid alterations in physical, psychological, and social development (Casey et al., 2008). As a big challenge in social adjustment and feelings of vulnerability associated with the relatively immature cognitive control performance, it may elicit a higher incidence of affective disorders and addiction among adolescents (Steinberg, 2005). Internet addiction (IA), as a new disorder, has been a public issue with fast developing of internet in recent years. Data from the China Youth Internet Association (announced on February 2, 2010) showed that the incidence of IA for Chinese urban youths is about 14% with the total number of 24 million (Yuan et al., 2011). IA consists of three subtypes: Internet gaming disorder (IGD), sexual preoccupations, and email/text messaging (Block, 2007). In China, the most important subtype of IA is IGD, and appendix of Diagnostic and Statistical Manual of Mental Disorders (5th Ed., DSM-5) also includes IGD, which emphasized that more research is needed to explore its clinical relevance and underlying neural mechanisms (Brand et al., 2014). The problem of IA drew much focus from education experts, psychologists and psychiatrists, so a lot of researches were performed on IA to investigate its brain mechanism and behavioral intervention (Ko et al., 2009, 2013a; Ding et al., 2013). However, currently the mechanism of IA is not clear and there is no standardized treatment for IGD available.

Adolescents with IGD spend ever-increasing amounts of time for online activities, leading to social withdrawal, self-neglect, poor diet and family problems (Murali and George, 2007; Young, 2007; Kim and Haridakis, 2009). It has been regarded as a behavioral disorder like pathological gambling (King et al., 2012), sexual activity (Holden, 2001), for they shared similar clinical symptoms including excessive use, withdrawal, tolerance, and negative repercussions (Beard and Wolf, 2001). A research showed that cognitive control has been altered in participants with heavy gamblers relative to controls (Toneatto et al., 1997), which suggested that addiction may compromise the cognitive control function. Cao et al. reported a specific relationship between cognitive control and IA by using questionnaires, and the IGD subjects exhibited more impulsivity than control group (Cao et al., 2007).

Cognitive control refers to the ability to control one’s own actions, behavior, and even thoughts (Cools and D’Esposito, 2011), as well as the capacity to flexibly adapt thoughts and behavior to current goals by selecting and integrating relevant information from the environment (Blasi et al., 2006). Studies have revealed that the anterior cingulate cortex (ACC) was involved in value assessment for cues pictures, emotional responses induced by craving, and the dorsal lateral prefrontal cortex (DLPFC) participated in cognitive processing for expecting reward and response after received reward (Sun et al., 2012; Brand et al., 2014; Ding et al., 2014). Several studies found cognitive control ability of IGD subjects was altered, for they showed more response errors and longer reaction time (RT) in Stroop task and Go-Nogo tasks in comparison with controls. For Stroop task, the response time, and response errors or mean error rates during the incongruent condition has been key indicators to assessing cognitive control function in IGD studies (Dong et al., 2013a, 2014; Yuan et al., 2013a). In details, Yuan et al. observed that both groups showed significant Stroop effect, where the RT was longer during the incongruent than the congruent condition. The IGD group committed more errors than the control group during the incongruent condition (Yuan et al., 2013a,b; Xing et al., 2014). Dong et al. consistently reported that the IGD group showed reduced efficiency of response-inhibition processes relative to healthy controls, for they demonstrated a non-significant trend for longer RTs (Dong et al., 2012, 2013a,b, 2014). On the other side, Go-Nogo and/or Go-stop tasks have been used to study behavioral characteristics of IGD. One study found that the scores of participants with IGD were significantly correlated with the number of failed no-go trials, suggesting that the low gaming-related inhibition or high impulsivity in IGD group (van Holst et al., 2012). Li et al. reported that the percentage of successfully inhibited responses was significantly lower in IA group than controls in a Go-stop task, which further supported that response inhibition in IA adolescents was impaired (Li et al., 2014).

Furthermore, many studies with neuroimaging and electrophysiological techniques investigated brain changes and cognitive control function in IGD. Dong et al. found that greater activity in the anterior (and also posterior) cingulate cortex for the interference condition of Stroop paradigm in participants with IGD compared with control subjects (Dong et al., 2012). Increased brain activities in the inferior frontal cortex and ACC may be implicated in altered cognitive control ability (Dong et al., 2013a). Yuan et al. also found that cortical thickness and amplitude of low frequency fluctuation (ALFF) values of prefrontal cortex correlated with the Stroop effect, providing brain image evidence for dysfunction in cognitive control performance of IGD. An Event-related potential (ERP) study also found that IGD group demonstrated lower NoGo-N2 amplitude, higher NoGo-P3 amplitude, and longer NoGo-P3 peak latency, indicating that they engaged in more cognitive endeavors, less efficiency in information processing, and lower impulse control than their normal peers (Dong et al., 2010). Another ERP study reported that people with IGD showed reduced medial frontal negativity (MFN) deflection in incongruent conditions than controls, which implied impaired cognitive control in IGD (Dong et al., 2011). However, few researches focused on the relationship between alteration of gray matter volume (GMV) and cognitive control ability in IGD.

The main purposes of the present study were: (1) to investigate cognitive control function with color-word Stroop task; (2) to explore stops aaddiction alteration of brain GMV using voxel-based morphometry (VBM) method; (3) to investigate the correlation between neuroimaging measures and behavioral performances in IGD. Based on the published literature on IGD, we hypothesized that IGD participants will show compromised performance for Stroop task and reduced GMV of the prefrontal cortex. Moreover, the prefrontal cortex GMV will be negatively correlated with the Stroop task performance in IGD individuals.

## Materials and Methods

All research procedures were approved by the First Affiliated Hospital of Medical College in Xi’an Jiaotong University Subcommittee on Human Studies and were conducted in accordance with the Declaration of Helsinki.

### Subjects

Twenty-eight college students with IGD were recruited in our study based on the criteria of the modified Young Diagnostic Questionnaire for Internet addiction (YDQ) by Beard and Wolf (Young, 1998; Beard and Wolf, 2001). Young suggested that respondents who answered five or more “yes” for the eight questions were considered to be an internet dependent user (Young, 1998). Beard and Wolf modified the YDQ criteria (Beard and Wolf, 2001), proposed that respondents who answered “yes” to questions 1 to 5 and at least to any one of the remaining three questions were classified as suffering from IA, which was used for screening subjects for the present study. We asked the subjects to recall their life-style when they were initially addicted to the internet, which was a retrospective measure for the addiction is a gradual process and we planned to explore linear changes of the brain structure. We retested them with the YDQ criteria modified by Beard and Wolf (Brand et al., 2014) to verify that they qualified for IA diagnosis. By communicating with their parents via telephone we confirmed the reliability of the self-reports from the IGD subjects. We also confirmed this information from their roommates and classmates that if they often played internet game till late night so that disturbing others’ life. Twenty-eight age and gender matched (*p* > 0.05) healthy controls without personal or family history of psychiatric disorders were also recruited in our study. In order to ensure that the healthy controls were not suffering from IGD, they were administered by modified YDQ for Internet addiction of Beard and Wolf. All recruited participants were native Chinese speakers, right-handed. Urine test was performed for all subjects to exclude substance abuse before magnetic resonance imaging (MRI) scanning. Exclusion criteria for both groups were (1) neurological disorders or physical illness, including brain tumor, hepatitis, or epilepsy assessed by clinical evaluations and medical records; (2) alcohol, nicotine or drug abuse; and (3) pregnancy or menstrual period in women; Written consent forms were obtained by all the patients and controls. More detailed demographic information was given in Table 1.

**Table 1:** **Demographics of internet gaming disorder and control groups**.

Items	IGD	Control	*p* value
Age (years)	18.8±1.33	19.3±2.56	>0.05
Gender	M18,F10	M20,F8	>0.05
Education (years)	12.18±0.48	12.2±0.56	>0.05
Hours of internet use(/day)	7.9±1.35	2.6±0.96	*
Days of internet use(/week)	5.35±1.31	2±0.71	*
Internet addiction test(IAT)	65.3±11.31	30.4±5.85	*
Duration of online gaming (years)	5.25±2.15	2.81±1.38	*

*p < 0.005.

### MRI Data Acquisition

Brain imaging scan was performed on a 3T GE scanner at imaging center of Xi’an Jiontong university first affiliated hospital. A standard birdcage head coil and restraining foam pads were used to minimize head motion and protect hearing. The axial 3D T1-weighted images were obtained with a spoiled gradient recall sequence and the following parameters: repetition time (TR) = 8.5 ms; echo time (TE) = 3.4 ms; flip angle (FA) = 12°; field of view (FOV) = 240 × 240 mm^2^; data matrix = 240 × 240; slices = 140; voxel size = 1 × 1 × 1 mm.

### MRI Data Analysis

MRI structure data was analyzed with FSL-VBM (Douaud et al., 2007),^1^ an optimized VBM protocol (Good et al., 2001) of FSL (Smith et al., 2004). First, structural images were brain-extracted and segmented gray matter was registered to the MNI 152 standard space using non-linear registration (Andersson et al., 2007). The resulting images were averaged and flipped along *x*-axis to create a left-right symmetric, study-specific gray matter template. Second, all native gray matter images were non-linearly registered to this study-specific template and “modulated” to correct for local expansion (or contraction) due to the non-linear component of the spatial transformation. The modulated gray matter images were then smoothed with an isotropic Gaussian kernel with a sigma of 3 mm. Finally, voxel wise GLM was applied by correcting for multiple comparisons across space. Regional structure in gray matter were assessed by permutation-based non-parametric testing (5000 times) (Nichols and Holmes, 2002).

### Behavioral Data Collection

Color-word Stroop task was implemented by E-prime 2.0 software. This task included a block design with three conditions, i.e., congruent, incongruent and rest. Red, Blue, and Green, three words were displayed in three colors (red, blue and green) as the congruent and incongruent stimuli. During rest, the subjects just focused their eyes on the cross displayed at the center of the screen. We designed two runs with different sequences of congruent and incongruent blocks (Xing et al., 2014). We tested participants individually in a quiet room and the participants kept a calm state of mind. Each of them was instructed to respond to the displayed color as fast as possible by pressing a button on a Serial Response Box TM with the right hand. The index, middle, and ring finger of right hand corresponding to red, blue, and green were used to press button respectively. The behavior data was collected two or three days before MRI scanning after practice.

### The Process of Correlation Analysis

Analysis of covariance (ANCOVA) was employed with age, gender effects and total intracranial volume as covariates. We used a *post hoc* correlation analysis to investigate relationship between GMV and behavioral performances in IGD group, and response errors and response time for incongruent condition of color-word Stroop task were employed to be the factors of correlation respectively of IGD group.

## Results

Our results showed that the average ages of IGD and control group were 18.8 ± 1.33 and 19.3 ± 2.56 years old, and there is no statistical difference between them (*p* > 0.05). According to their self-report of Internet use, the time spending by IGD adolescents per day and per week were more than the control group (*p* < 0.005).The IGD individuals spent longer duration of time on online gaming (*p* < 0.005) (Table 1).

### Behavioral Results

A significant Stroop effect were observed in both group, where the RT was longer for the incongruent relative to the congruent condition (IGD group: 628.24 ± 59.20 vs. 549.38 ± 44.17 and control group: 707.52 ± 66.43 vs. 581.97 ± 39.35; *p* < 0.005). The IGD group committed more errors than the control group during the incongruent condition (IGD group: 8.67 ± 5.41 vs. control group: 6.64 ± 3.65; *p* < 0.05), and the response delay (RD) measured by RT during the incongruent condition minus congruent conditions was significantly different between these two groups (IGD group: 78.87 ± 45.38 vs. control group :125.56 ± 49.20; *p* < 0.05) (Table 2).

**Table 2:** **Behavioral results for internet gaming disorder and control groups**.

Items	IGD		Control	
	Congruent	Incongruent	Congruent	Incongruentn
RT (ms)	549.38±44.17	628.24±59.20	581.97±39.35	707.52±66.43
Errors	3.21±2.38	8.67±5.41	3±2.04	6.64±3.65
RD (ms)	78.87±45.38		125.56±49.20	

*Form: Mean ± standard deviation*.

### Brain Imaging Results

**Figure 1:**
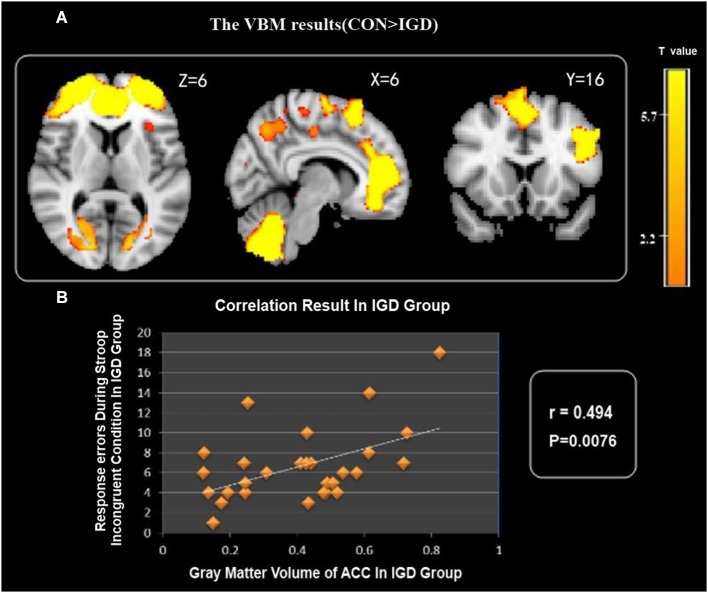
**(A)** IGD group showed reduced gray matter volume (GMV) in the bilateral ACC, precuneus, SMA, superior parietal cortex, cerebellum, left DLPFC and left insula. **(B)** Correlation between GMV of ACC and Stroop task response errors during incongruent condition in IGD group.

VBM comparison indicated decreased GMV in several brain areas, i.e., the bilateral ACC, precuneus, supplementary motor area (SMA), superior parietal cortex, left DLPFC, left insula and bilateral cerebellum in the IGD group compared with the control group (Figure 1).

### Correlation Analysis Results

Correlation analysis showed that the GMV of the ACC negatively correlated with Stroop task response errors for incongruent condition in the IGD group (Figure 1), but there was no statistical correlation between the GMV and RT for incongruent condition in the IGD group.

## Discussion

Adolescence is a period with significant changes in both the social landscape and brain development, which is also a time with higher incidence of affective and addiction problems (Casey et al., 2008). Many scientist in Asia have reported that IGD became a public health problems in teenagers and youth (Ko et al., 2007; Park et al., 2008). It is difficult to have a valid therapy based on the unclear mechanism of IA. The brain structure changes and cognitive control deficits were observed in IGD adolescents. However, investigating relationship between brain structure and cognitive control in IGD is critical for developing possible intervention for this disorder. In the present study, reduced cognitive control ability and abnormal brain GMV in the IGD adolescents were observed compared with the control group, and more importantly, there was a negative correlation between the GMV of ACC and response errors for incongruent condition in the color-word Stroop task in IGD group.

### The Alteration of Behavior Changes and Gray Matter Volume in IGD Group

In order to verify impaired cognitive control ability in adolescents with IGD, a color-word Stroop task was used in the current study. Consistent with previous findings (Dong et al., 2011, 2013a; Yuan et al., 2013a,b), the IGD group committed more errors than the control group during the incongruent condition, which demonstrated that adolescents with IGD showed impaired cognitive control ability, as measured by the color-word Stroop test. The result that the RT during incongruent condition and RD of IGD group were shorter than control group may be imply that IGD subjects showed a different reaction pattern relative to controls, and they responded fast but taking the risk of making more errors, which was clearly a change in the response strategy. The study also found that the GMV of the ACC, DLPFC, precuneus, SMA, superior parietal cortex, insula, and cerebellum in the IGD group changed, which is in line with published IGD studies. Zhou and Weng et al. reported GMV reduction or abnormal activation in some brain areas in IGD subjects (Yuan et al., 2011; Zhou et al., 2011; Sun et al., 2012; Ko et al., 2013b; Weng et al., 2013). Although no study reported GMV of the precuneus decreased, fMRI study reported that the precuneus showed abnormal activation during cue-induced task in IGD subject (Ko et al., 2013a,b). The superior parietal cortex was found to be related with cognitive control (Durston et al., 2002, 2003; Ko et al., 2013a).

### The Relationship between Gray Matter Volume of ACC and Performance of Color-Word Stroop Task

The correlation between the GMV of ACC and response errors showed that less GMV of ACC in IGD group was associated with more response errors during the incongruent condition in color-word Stroop task, which is a promising finding for the present study. The role of the ACC in cognitive control was well established and has been reported in a number of fMRI studies on Stroop interference paradigm in normal participants. Botvinick et al. reported the ACC was involved with conflict-monitoring function, for the ACC was more active under conditions of high conflict (Botvinick et al., 1999). Another research of Angus W. MacDonald III discovered that the activity of ACC was dissociable from top–down control, and it played a consistent role in monitoring conflict during response period (MacDonald et al., 2000). The study of Kerns revealed that the conflict-related activity of ACC predicted both greater prefrontal cortex activity and adjustments in behavior, supporting a role of ACC in conflict monitoring and cognitive control (Kerns et al., 2004). Furthermore, Matsumoto demonstrated that the cognitive control recruited by the ACC may be “consequential” based on conflicts between evoked plans and concrete actions (Matsumoto and Tanaka, 2004). A large body of experimental evidence on numerous diseases has accrued to support the important function of ACC in cognitive control. Akio Soeda et al. studied traumatic brain injury (TBI) patients and found out that the decreased activation in the ACC may be associated with alteration in functional cerebral activity, which may reflect either cortical disinhibition attributable to disconnection or compensation for an inefficient cognitive process (Soeda et al., 2005). Abnormal activity of ACC has been found in many mental problems, including obsessive–compulsive disorder (OCD), attention deficit-hyperactivity disorder (ADHD), and major depressive disorder (MDD; Ursu et al., 2003; Liotti et al., 2005; Murali and George, 2007). Recent neuroimaging studies also found altered activation of the ACC in heroin- and opioid-dependent individuals in GO/NOGO paradigm (Forman et al., 2004), suggesting the ACC is a key area in response inhibition (Fu et al., 2008). The research on cocaine users confirmed activity of ACC in inhibitory control (Kaufman et al., 2003; Goldstein et al., 2007, 2009). A Magnetic Resonance Spectroscopy (MRS) study on nicotine dependence showed that glutamate + glutamine (Glx) levels reduced in ACC, indicating the ACC was involved in cognitive control by modulating behavior (Wheelock et al., 2014). In a word, the ACC is important for cognitive control ability. The ACC’s structural abnormalities and dysfunction in IGD have been reported in previous studies. The VBM results of Zhou et al. showed that the GMV of ACC decreased in IGD compared with controls (Yuan et al., 2011; Zhou et al., 2011). Many researches on IGD indicated that the ACC participated in the cognitive control, such as inhibitory control, error monitoring, and decision making (Dong et al., 2012, 2013a,b).

## Conclusion

In the present study we found GMVs reduced in the ACC and other brain regions, as well as behavior pattern altered in cognitive control processing, which is consistent with published brain image studies on IGD and other addiction, suggesting IGD compromised both behavioral activity and neural structure in adolescents with IGD. Furthermore, we also found that ACC volume negatively correlated with incongruent response errors for Stroop paradigm, indicating a totally different response pattern in IGD individuals and its negative impacts on brain structure in adolescents.

## Conflict of Interest Statement

The authors declare that the research was conducted in the absence of any commercial or financial relationships that could be construed as a potential conflict of interest.

## Footnotes

^1^http://fsl.fmrib.ox.ac.uk/fsl/fslwiki/FSLVBM
